# Salivary Fluoride Bioavailability after Brushing with Brazilian Red Propolis Dentifrice: A Clinical Study

**DOI:** 10.1155/2022/6148137

**Published:** 2022-01-31

**Authors:** Francisco Josimar Girão Júnior, Lidia Audrey Rocha Valadas, Peter Bottenberg, Mara Assef Leitão Lotif, Edilson Martins Rodrigues Neto, Said Gonçalves da Cruz Fonseca, Mary Anne Medeiros Bandeira, Aldo Squassi, Thereza Cristina Farias Botelho Dantas, Nara Juliana Custódio de Sena, Marta Maria de França Fonteles

**Affiliations:** ^1^Pharmacology Postgraduate Program, Federal University of Ceará, Fortaleza, Brazil; ^2^Cátedra de Odontología Preventiva y Comunitaria, Facultad de Odontologtía, Universidad de Buenos Aires, Buenos Aires, Argentina; ^3^Department of Oral Health Sciences, Université Libre de Bruxelles (Free University of Brussels), Brussel, Belgium; ^4^Postgraduate Program in Drug Development, Federal University of Ceará, Fortaleza, Brazil; ^5^Paulo Picanço College of Dentistry, Fortaleza, Brazil; ^6^Christus University Center, Fortaleza, Brazil

## Abstract

**Introduction:**

Fluoride plays an important role in the control of dental caries, and currently new dentifrices are being associated with natural products.

**Objective:**

This study aimed to evaluate the availability of fluoride in saliva samples after using a dentifrice incorporated with Brazilian red propolis (BRP, INPI Patent no. BR1020170110974) and to compare it to a conventional fluoridated dentifrice in healthy participants.

**Methods:**

This study was conducted implementing a double-blind, randomized, controlled, and crossover design. Saliva samples of participants were collected at the following time points: 0 at baseline and 5, 15, 30, 45, and 60 minutes after brushing with each dentifrice. Salivary fluoride concentrations showed no statistically significant difference when comparing the two treatments (*p* > 0.05). All available fluoride concentrations in saliva decreased after one hour, with no significant difference between BRP and conventional fluoridated dentifrice treatment samples (*p* > 0.05).

**Results:**

The results showed that there was no difference between the analyzed fluoride concentrations 1 hour after brushing with the different dentifrices.

**Conclusions:**

The results of this study suggest that the propolis incorporated in the dentifrice did not interfere with the kinetics and bioavailability of the fluoride ion in saliva samples, enabling its integration with the pharmaceutical formula and thereby promoting its anti-inflammatory and antimicrobial benefits without compromising the anticaries activity of the formulation.

## 1. Introduction

Dental caries is one of the main oral diseases that affect the world population, and it is directly related to dental biofilm and diet. A demineralization of dental hard tissues occurs in dental caries; involved in this process are the dental structure, biofilm, sugar intake, and salivary factors. Protective factors such as use of fluoride and dental biofilm control are essential to prevent this disease [1].

Fluoride plays an important role in the prevention and control of dental caries through the well-understood process in which it reduces tooth demineralization and favors remineralization [2, 3]. This is associated with the decline in the prevalence of lesions in the world population in the last 50 years through implementing public water fluoridation systems and using fluoride products for personal and professional care. However, fluoridated dentifrices are by far the most important vehicle for providing fluoride [4].

The effect of fluoride on the control of dental caries is local and depends on constant maintenance in the oral cavity so that it can interfere with the process of developing lesions [2]. It is considered the most important therapeutic substance added to dentifrices, considerably increasing the effect of mechanical toothbrushing on the control of tooth decay [3]. In addition to fluoride, dentifrices can contain other substances with therapeutic properties in order to reduce hypersensitivity and dental calculus formation and control biofilm [1]. These substances can be of natural or industrial origin. Compounds of natural origin have been used in formulations in searching for products with therapeutic activity, low cost or local availability, biocompatibility, and (reputedly) lower toxicity [5].

Brazilian red propolis (BRP) stands out among the natural products, which have been studied for developing new dental formulations, especially in dentifrices and mouthwashes [5–9]. It is considered a promising source for developing new products based on natural raw materials as a result of its antimicrobial, antifungal, antioxidant, antitumor, anti-inflammatory, cytotoxic, antiulcer, immunomodulatory, and cardioprotective properties [10–13]. In addition, BRP has demonstrated antibiofilm and anticariogenic activity in vitro and in vivo [5–9].

Thus, a fluoride dentifrice incorporated with BRP 1% was developed because of the therapeutic properties of BRP, mainly antimicrobial, demonstrated by several studies [5, 10–13]. This dentifrice has demonstrated clinical and microbiological efficacy in orthodontic patients with gingivitis [5]. In designing dentifrices with therapeutically active compounds, it must be made sure that there is no unwanted interaction with fluoride [14]. As the fluoride present in dentifrices is important in the process of preventing and controlling dental caries, it is important to assess not only in laboratory conditions whether there is the maintenance of the appropriate levels of fluoride in saliva when using dentifrice incorporated with BRP.

In view of the interaction between fluoride and BRP, it is believed that there may be an increase in anticariogenic effects, generating greater protection against the demineralization process and greater dental mineralization. Thus, the beneficial interaction between propolis and fluoride has already been studied through other pharmaceutical forms such as cement glass ionomer, mouthwash, and gel, being verified that there was no pharmaceutical incompatibility [15–17].

In view of the above, the aim of this study was to evaluate the availability of fluoride in the saliva of healthy individuals after brushing teeth with the BRP 1% fluoride dentifrice compared with a conventional fluoride dentifrice.

## 2. Materials and Methods

### 2.1. Type of Study and Ethical Aspects

This is a randomized, double-blind, controlled, and crossover clinical trial carried out after approval by the research ethics committee under the opinion number 3,358,397. The study methodology followed the CONSORT checklist and it is registered in the Brazilian Registry of Clinical Trials (REBEC RBR-53pppb) under the Universal Trial Number U1111-1245-8255.

### 2.2. Preparation of Dentifrice

The dentifrices of the study were prepared in the pharmaceutics laboratory of the Pharmacy course at the Federal University of Ceará, Brazil, at a concentration of 1,500 ppm F in the form of monofluorophosphate (MFP) and containing calcium carbonate (CaCO_3_) as an abrasive agent.

For the preparation of the BRP dentifrice, in the basic fluoridated dentifrice formulation, BRP extract from the region of Marechal Deodoro, AL, Brazil, was incorporated (south latitude 9° 44.555′, west latitude 35° 52.080′ and altitude of 18.1 m above sea level), which has a geographical indication of the extract by the National Institute of Industrial Property. The concentration of 1% was used, previously studied in vitro and in vivo with the same dentifrice. The BRP dentifrice is patented under reference number BR1020170110974A2.

### 2.3. Examiners and Procedures for Selecting Participants

The participant selection and the clinical trial were performed in the School of Dentistry at Federal University of Ceará, Brazil, by the responsible researcher and two dental surgeon examiners. The sample size was calculated based on similar studies with a 95% confidence interval, considering a power of analysis of 0.8 at a significance level of *p*=0.05, resulting in a minimum of 7 individuals in order to produce reliable data [18, 19].

The patients were initially explained the objectives and all ethical aspects of the study. Individuals interested in participating were treated individually, where personal and general health data were collected and a preliminary clinical screening test was carried out in which the general oral health condition and the index of dental caries were evaluated using the ICDAS II (International Caries Detection and Assessment System) method by previously two calibrated examiners (*κ* = 0.76). Inclusion criteria were as follows: aged between 12 and 18 years; ICDAS II 0; being right-handed; being healthy; and not using any medication. Individuals who met any of the following exclusion criteria were excluded from the study: presence of periodontal disease; systemic diseases or a history of allergies; licit/illicit drug users; patients with oral prostheses; orthodontic appliance users; presence of less than 10 dental elements per dental arch; pregnancy; and individuals with flowrate less than ≤0.2 mL.

Thus, after informing potential participants, eight eligible individuals who did not meet any exclusion criteria signed the free and informed consent form at the end of the screening and were included in the study. The participants filled out an anamnesis form one week before starting treatments and received periodontal treatment through scaling to remove supragingival biofilm and prophylaxis with pumice and water.

### 2.4. Clinical Phase

In this crossover study, the eight participants included were randomized into two groups in order to define which dentifrice would be used in brushing in each of the two periods.

**Figure 1:**
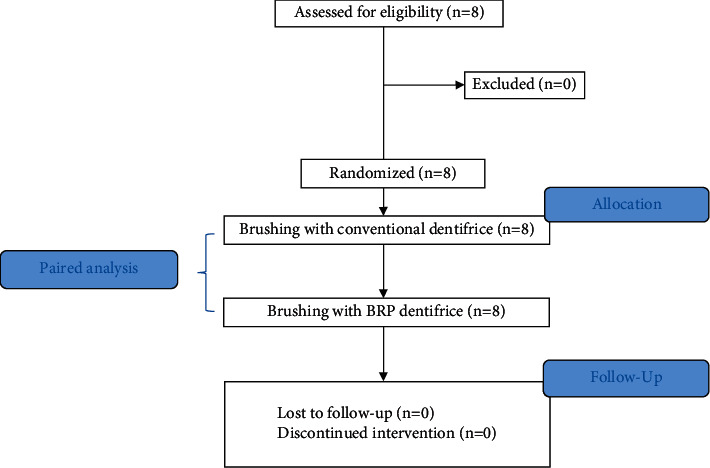
Study design flowchart.

At start, they received a nonfluoridated dentifrice to be used for brushing in a washout period, which consisted of its use for three days to avoid interference in the analysis [20]. All participants received instructions on brushing technique. After each washout period, with at least 2 hours of fasting, the participants brushed with 1 g of dentifrice, weighted in a precision balance, for 1 minute according to their allocation group, followed by rinsing with 10 mL of distilled water for 10 seconds. There were six saliva collections performed for the analysis in the two periods at different time points (0—baseline, and after tooth brushing with the dentifrice at 5, 15, 30, 45, and 60 minutes). The study design is represented in Figure 1.

### 2.5. Saliva Collection and pH Analysis

Approximately 2 mL of unstimulated saliva was collected from each participant at different time points for the analysis through a Pasteur pipette and stored in sterile microtubes (Eppendorf®). The pH was measured at the time of each collection using pH tapes (Macherey-Nagel, Düren, Germany). Next, the samples were stored under cooling at −80°C to be analyzed for fluoride content the next day.

### 2.6. Analysis of Total, Soluble, and Ionic Fluoride

The method used was proposed by Cury [21] and modified by Orth et al. [22] for saliva samples. An ion-specific fluoride electrode (Orion 96-09, Orion Research Inc., Beverly, MA, USA) was used. Total fluoride (TF), total soluble fluoride (TSF), and ionic fluoride (IF) were analyzed. The electrode was calibrated with fluoride standards before analysing the samples, ranging from 0.5 to 32.0 ppm F, prepared in triplicate by serial dilution of a 1000 ppm NaF stock solution (ANALYSER no. 110902, Analyser Comércio e Indústria Ltda., Paraná, Brazil). The analysis of the dentifrices was performed according to the method proposed by Cury [21]. The fluoride concentration in the samples was calculated from the linear regression of the calibration curves obtained with standard fluoride concentrations. Millivolt potentials were converted to ppm of fluoride using a standard curve with a correlation coefficient of *r* ≥ 0.99.

**Figure 2:**
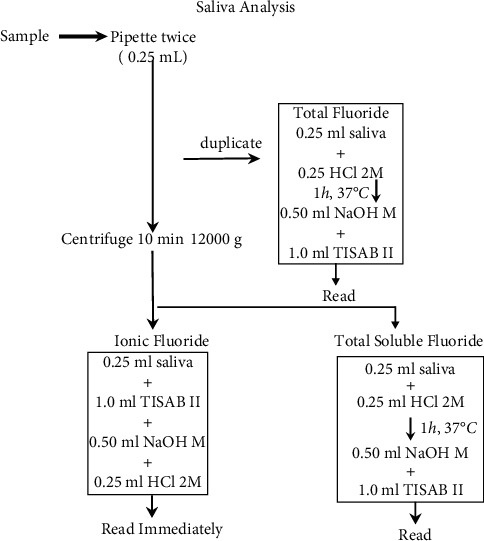
Saliva analysis flowchart. HCl = hydrochloric acid, NaOH = sodium hydroxide, and TISAB II = total ionic strength adjustment buffer II.

All samples were read in duplicate. Next, 0.25 mL of 2 mol·L^−1^ hydrochloric acid was added to 0.25 mL of each saliva sample for the TF (whole saliva) and TSF (centrifuged sample) measurement, and the samples were kept for 1 hour at 37°C in an oven. Then, neutralization was performed with 0.5 mL of 1 mol·L^−1^ sodium hydroxide and the samples were buffered with 1 mL of TISAB II (total ionic strength adjustment buffer). The procedure for IF was performed by direct reading of the centrifuged saliva sample added by the same reagents mentioned above. The procedures performed are illustrated in Figure 2.

### 2.7. Statistical Method

The results were initially analyzed by the Kolmogorov–Smirnov test to verify the normality of the distribution, demonstrating that the sample is predominantly not normally distributed for the fluoride samples and predominantly normally distributed for pH. This way, the median and interquartile range were calculated for descriptive statistics. The comparisons between the results at different time points were performed using the *t*-test for pH and the Mann–Whitney test, Friedman test, and nonparametric ANOVA for fluoride analysis.

In all cases, the *α*-probability of type I error (level of significance) was set at 0.05 (5%), with a two-tailed *p* value less than 0.05 being considered statistically significant. GraphPad Prism® software version 5.00 for Windows® (GraphPad Software, San Diego, CA, USA, 2007) was used.

## 3. Results

All participants completed the study with 12 samples collected from each, resulting in 96 samples. The basal fluoride content in saliva varied in the eight participants in concentrations from 0.009 to 0.040 ppm.

The conventional dentifrice presented mean values in mg/mL of 1462.0 for TF, 1421.2 for TSF, and 162.6 for IF while the BRP dentifrice presented 1488.8 for TF, 1039.1 for TSF, and 351 for IF.

**Figure 3:**
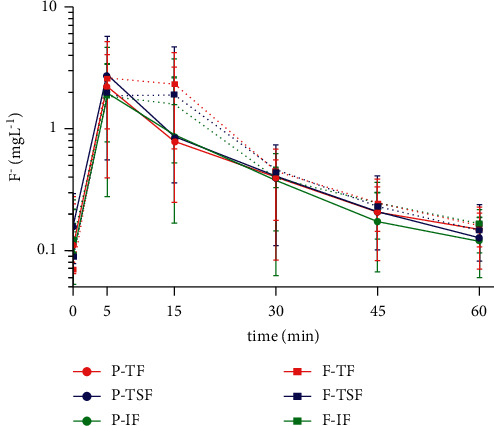
TF, TSF, and IF concentration in saliva after brushing with dentifrice at different time points. Mean (±SD, *n* = 8) of salivary F concentration (mg/ml) according to the time (minutes) after brushing with both dentifrices is given. Vertical bars show the standard deviation. *P* = fluoride dentifrice containing BRP, F = control fluoride dentifrice, TF = total fluoride, TSF = total soluble fluoride, and IF = ionic fluoride.

The values for fluoride concentrations in the participants' saliva samples after brushing with each dentifrice are shown in Figure 3.

Table 1 expresses the groups individual values of F concentration over time.

The TF, TSF, and IF concentrations in saliva significantly increased after brushing with conventional dentifrice, BRP dentifrice, or control, reaching maximum concentrations in the first five minutes of the test and gradually decreasing over time, with no significant difference between the fluoride forms within these groups (*p* > 0.05).

**Figure 4:**
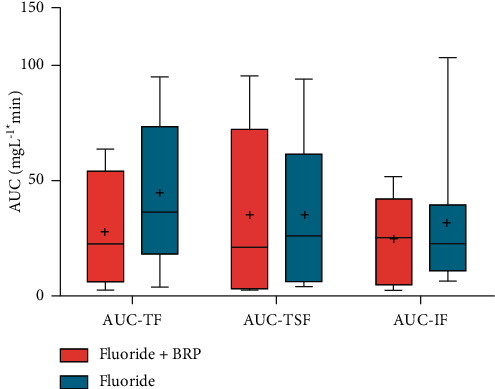
Area under the curve of the TF, TSF, and IF concentrations in saliva after the use of dentifrice. Mean AUC of the salivary F concentration versus time (mg F/mL.min) according to the treatments (*n* = 8) is given. Vertical bars show the standard deviation. No significant differences were seen between TF/TSF/IF. There was statistically significant difference between TF-P and TF-F (two-way ANOVA) (*p* < 0.05). AUC = area under the curve TF = total fluoride, TSF = total soluble fluoride, and IF = ionic fluoride.

Figure 4 shows the values for the area under the concentration curve (mgL^−1^∗ min) of the different fluoride forms present in the saliva considering the use of each dentifrice. Significant statistical difference is shown only when comparing the TF measurements between the two dentifrices (*p*=0.97, Friedman test).

**Figure 5:**
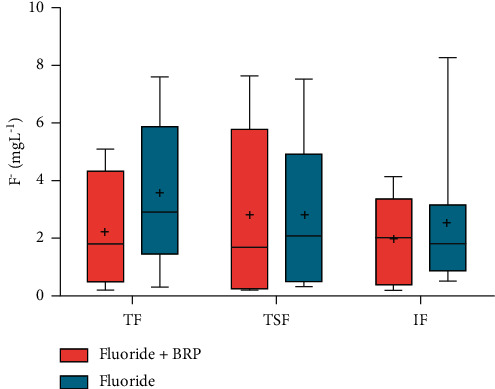
Maximum TF, TSF, and IF concentrations in saliva after using dentifrice. Mean (±SD, *n* = 8) of salivary F concentration (mg/ml) according to the treatments (*n* = 8) is given. Vertical bars show the standard deviation. TF = total fluoride, TSF = total soluble fluoride, and IF = ionic fluoride.

Figure 5 shows the comparisons between the maximum TF, TSF, and IF concentrations (mgL^−1^) found in the samples after using each dentifrice. It was observed that the concentrations of the fluoride forms for the BRP dentifrice varied from 0.19 to 7.64 and from 0.33 to 8.26 for the conventional fluoride dentifrice, with no statistically significant difference between the three groups (*p* = 0.52, Friedman test).

Regarding pH, there was no statistical difference between the saliva samples analyzed at any time (*p* > 0.05), with averages of 7,104 for the BRP dentifrice and 7,083 for the conventional fluoridated dentifrice (*t*-test).

## 4. Discussion

Some studies have evaluated the fluoride concentration after brushing using a similar methodology, suggesting that a fluoride deposit occurs in oral fluids soon after the use of these materials [2, 18–20, 23]. For this purpose, the ion-selective electrode (ISE) has several advantages such as ease of use and method effectiveness.

The development of new dental formulations with antimicrobial activity is increasing every day due to bacterial resistance, toxicity, and high costs of available materials, thereby constituting interesting alternative products with real possibilities of lesser adverse effects and low cost [24]. The most used antimicrobial agents in dentistry are chlorhexidine and triclosan, for which studies point to adverse effects [25, 26]. One previous clinical trial study with the same BRP dentifrice reported a better decrease of gingival bleeding index and salivary bacteria counts when compared with a regular dentifrice, suggesting a new alternative to control biofilm [5].

The TF, TSF, and IF concentrations in saliva significantly increased after brushing with dentifrice in this study, reaching maximum concentrations in the first five minutes of the test, gradually decreasing over time, with no significant difference between groups when comparing the different time points. Similar data have been found in studies such as those by Naumova et al. [27], Staun Larsen et al. [28], and Bezerra et al. [29], showing an increase in concentration in the first samples collected, followed by a gradual decrease inversely proportional to the elapsed time. In addition, the study conducted by Naumova et al. [27] presented interindividual variability in fluoride concentrations, similar to those found in the present study.

It is known that the addition of components to dentifrices can interfere with the action of fluoride; however, there was no decrease in the available concentration after 1 hour of brushing with the addition of BRP extract in the present formulation. It was verified that the incorporation of 1% propolis extract in the glass ionomer cement increased the fluoride release, not generating pharmaceutical incompatibility. The hypothesis raised was that propolis could favor the release of fluorine from the polymeric matrix [17]. It was observed that there was no significant difference between the concentrations for any form of fluoride analyzed when comparing the TF, TSF, and IF concentrations in saliva at 1h in the same patient after using each dentifrice, demonstrating that the fluoride concentration was maintained with the incorporation of BRP to the dentifrice.

Considering the different treatments between groups, the maximum concentrations in mgL^−1^ of the different forms of fluoride also did not show a statistically significant difference. A statistically significant difference for the AUC (mgL^−1^ ∗ min) was only observed for TF. There was no statistically significant difference for TSF and IF.

Unlike NaF, fluoride in the form of MFP does not start its action immediately when released in saliva, needing to be dissociated through hydrolysis to become available. The hydrolysis of the MFP is pH-dependent, occurring better at a pH between 7.2 and 8.6 in saliva, which may justify the values found, since pH levels similar to these were found [30].

The saliva collection was established at 0, 5, 15, 30, 45, and 60 minutes to detect the moments of higher fluoride concentration in the saliva, as well as the moments when there was an eventual statistical difference between the different dentifrices. The concentrations in this study ranged from 0.009 to 8.261 ppm F. The fluoride concentration in saliva was verified within 60 minutes, because after this period of brushing with fluoridated dentifrice, it is reported that there is a drastic drop in the concentration [2, 31]. The same is observed when fluoridated mouthwash is used [30]. Some data indicate that fluoride acts on the enamel even in low concentrations of 0.1 ppm in oral fluids and can interfere with the cariogenic process [31].

In addition to the effectiveness of a product, acceptance by patients is essential. Participants' acceptance of the dentifrice incorporated with BRP used in this study was evaluated in a previous study carried out by Amaral Silva et al. [32], in which they obtained excellent results in terms of taste, odor, and cleanliness.

Thus, it is believed that this pharmaceutical formulation is indicated for patients who need chemical control of biofilm in addition to mechanical control, such as those with dental plaque retentive factors. Furthermore, it is a viable and safe dentifrice for patients, has proven clinical efficacy, and maintains bioavailable fluoride in saliva [5, 32].

Brushing with BRP dentifrice can be an important strategy, providing chemical and mechanical control of oral biofilm through a single low-cost product. Thus, this product could be a pharmacoeconomically viable alternative as an adjuvant in the prevention of diseases related to bacterial biofilm, which reinforces the importance of clinical studies about it.

As limitations of this study, there is a need to carry out studies with the BRP dentifrice comparing the concentration and bioavailability of fluoride in other biological environments; more participants and for a longer period are important.

## 5. Conclusion

There was no difference between the analyzed fluoride concentrations 1 hour after brushing with the different dentifrices. Thus, the results of this study suggest that the BRP incorporated into the dentifrice did not interfere with the kinetics and the fluoride ion availability in the saliva samples, enabling its integration with the pharmaceutical formula and promoting its anti-inflammatory and antimicrobial benefits without compromising the anticaries activity of the formulation.

## Figures and Tables

**Table 1:** F concentration means in mg/mL over time.

Time in min/F concentration (min)	P-TF	P-TSF	P-IF	C-TF	C-TSF	C-IF
0	0.10	0.16	0.09	0.07	0.07	0.09
5	2.22	2.78	1.97	2.60	1.86	2.07
15	0.79	0.85	0.89	2.34	1.91	1.57
30	0.39	0.41	0.37	0.44	0.46	0.38
45	0.21	0.21	0.17	0.25	0.23	0.24
60	0.15	0.13	0.12	0.17	0.15	0.15

P: propolis dentifrice; C: conventional dentifrice; TF: total fluoride; TSF: total soluble fluoride; and IF: ionic fluoride.

## Data Availability

The datasets generated by the current study are available from the corresponding author upon reasonable request. The dissertation data used to support the findings of this study have been deposited in the UFC Institutional Repository (http://www.repositorio.ufc.br/handle/riufc/49649).
